# Case Report: Novel homozygous pathogenic variant of the *SPG20* gene causes the Troyer syndrome in China

**DOI:** 10.3389/fgene.2026.1842902

**Published:** 2026-07-02

**Authors:** Lina Zhu, Siqi Hu, Xinyang Jiang, Rujie Gu, Yongxia Wang, Shufang Zhang, Fujun Peng, Xiuwei Ma

**Affiliations:** 1 Senior Department of Pediatrics, The Seventh Medical Center of Chinese PLA General Hospital, Beijing, China; 2 Weifang Key Laboratory of Collaborative Innovation of Intelligent Diagnosis and Treatment and Molecular Diseases, School of Basic Medical Sciences, Shandong Second Medical University, Weifang, China

**Keywords:** hereditary spastic paraplegia, lipid droplet, rare disease, *SPG20*, Troyer syndrome

## Abstract

Hereditary spastic paraplegia (HSP) comprises a group of neurodegenerative disorders characterized by progressive spasticity of the lower limbs. Troyer syndrome (MIM #275900), an autosomal recessive form of complicated HSP, was initially described in the Old Order Amish population. The syndrome is associated with a spectrum of clinical manifestations, including spastic paraplegia, muscle atrophy, dysarthria, intellectual disability, and abnormal white matter on neuroimaging. The causative gene for Troyer syndrome has been identified as *SPG20*, which encodes the spartin protein. Spartin plays a pivotal role in lipid droplet degradation and mitochondrial function. Here, we report the clinical and molecular features of the second documented case of Troyer syndrome in China. The patient, a 5-year-8-month-old Han Chinese girl, presented with delayed psychomotor development, abnormal gait, and a history of febrile seizures. Whole-exome sequencing revealed a novel homozygous pathogenic variant, c.1734-1G>C, in *SPG20*, leading to a frameshift and the expression of a truncated protein. This variant was absent from multiple population databases of healthy individuals, and both parents were heterozygous carriers without clinical manifestations. Functional studies in cells transfected with *SPG20* constructs demonstrated that the variant spartin protein failed to localize to lipid droplets, leading to their accumulation. This functional impairment may contribute to the pathogenesis of Troyer syndrome. Our findings expand the variant spectrum of *SPG20* and provide further insight into the genotype–phenotype relationship in Troyer syndrome, highlighting the critical role of spartin in lipid metabolism and neuronal function.

## Introduction

Hereditary spastic paraplegia (HSP) is a group of neurodegenerative diseases with high clinical and genetic heterogeneity, and it is characterized by progressive spasticity of the lower limbs. The pathogenesis of HSP may involve axonal degeneration and demyelination of long descending axons to the lower limbs, although the precise mechanisms remain unclear. HSP can be classified according to the inheritance patterns as autosomal dominant (AD), autosomal recessive (AR), or X-linked recessive (XR), and it is categorized as pure or complicated based on the clinical manifestations. Complicated HSP is associated with variable manifestations and can form distinct syndromes.

Troyer syndrome (TRS; MIM #275900) is an AR complicated HSP. It was first reported in the Old Order Amish population by Cross and McKusick (1967). Clinical manifestations include spastic paraplegia of the lower limbs, distal limb muscle atrophy, prominent spastic dysarthria, pseudobulbar palsy, physical and intellectual retardation, skeletal deformities, mild cerebellar signs, choreoathetosis, and deafness. Brain MRI shows abnormal white matter, particularly in the periventricular regions of the temporal and parietal lobes. In 2002, Patel et al. performed linkage analysis in an AR complicated HSP family, locating the pathogenic gene within D13s1840–D13s1845 on chromosome 13q12.3, and confirmed *SPG20* as the causative gene (Patel et al., 2002). *SPG20* encodes spartin, which is widely expressed in both nervous and non-nervous tissues, and variants in this gene are causative of TRS (Patel et al., 2002). Recent studies indicate that spartin plays a pivotal role in lipid droplet (LD) degradation by delivering them to lysosomes via selective autophagy to mobilize triglycerides (Eastman et al., 2009; Wan et al., 2024; Chung et al., 2023). Spartin is also involved in mitochondrial protein import and bioenergetic metabolism. Dysfunction of spartin can lead to mitochondrial impairment, which is characterized by damage to complex-I of the mitochondrial respiratory chain and increased reactive oxygen species (Diquigiovanni et al., 2023; Cusen et al., 2022).

To date, only one previous case of Troyer syndrome has been reported in China (Liang et al., 2020). Here, we describe the second genetically confirmed Chinese patient with Troyer syndrome. Whole-exome sequencing (WES) identified a novel homozygous splice-site variant, c.1734-1G>C, in *SPG20*, causing a frameshift after amino acid 577 and the expression of a truncated protein of 598 amino acids (wild-type *SPG20*: 666 amino acids). We present the clinical features of the patient and the genotype–phenotype correlation. Functional studies demonstrated that the variant spartin protein in this case does not associate with the surface of LDs, indicating that spartin localization to LDs is significant in TRS.

## Methods

### Genomic DNA extraction

Two milliliters of peripheral blood from the patient, her parents, and sister were collected and sent to Kaiumph Medical Laboratory for analysis. Genomic DNA was extracted using the QIAamp DNA Blood Mini Kit (Qiagen), followed by WES.

### Whole-exome sequencing

For the patient, genomic DNA was used for library construction, whole-exome capture, and sequencing on the HiSeq X10 platform. Sequence alignment to the reference genome was performed using BWA software application. Variant calling was conducted with GATK, and annotation was performed using ANNOVAR software application. The raw data were filtered based on the minor allele frequency (MAF) in normal populations, variant function, and additional filtering criteria. Potential pathogenic variants were identified according to the patient’s clinical phenotype and American College of Medical Genetics and Genomics (ACMG) guidelines. Copy number variation (CNV) analysis was performed using an exon-level algorithm based on capture sequencing depth.

### RNA extraction and cDNA sequencing

Two milliliters of peripheral blood from the proband were collected for RNA extraction and cDNA sequencing at Kaiumph Medical Laboratory. Total RNA was extracted using a blood mRNA extraction kit. Specific PCR primers were designed flanking the c.1734-1G>C variant, and PCR amplification followed by sequencing was performed.

### DNA constructs, antibodies, and reagents

Wild-type (WT) and variant *SPG20* DNA sequences were cloned into the pCMV-Tag 3B vector. The variant *SPG20* construct was generated from the WT human *SPG20* cDNA using a standard PCR-based site-directed mutagenesis approach. Antibodies against Myc (2276S) and β-Actin (60008-1-Ig) were obtained from Cell Signaling Technology and Proteintech, respectively. Oleic acid (HY-N1446) was purchased from MCE.

### Human cell lines

HeLa cells were cultured in Dulbecco’s Modified Eagle Medium (DMEM; Thermo Fisher Scientific) supplemented with 10% fetal bovine serum (FSP500, ExCell), 100 U/mL penicillin, and 100 mg/mL streptomycin and incubated at 37 °C in a humidified 5% CO_2_ atmosphere. Plasmid transfection was performed using PEI (Sigma-Aldrich) according to the manufacturer’s instructions (Hu et al., 2019).

### Western blotting

For protein extraction, cells were lysed in RIPA buffer containing 1% NP-40, Triton X-100, 0.25% sodium deoxycholate (SDS), 150 mM NaCl, 20 mM Tris (pH 7.4), and 1 mM EDTA. Equal amounts of protein were separated by 12% SDS-PAGE (WB1103, Beijing Biotides Biotechnology Co., Ltd.) and transferred onto nitrocellulose membranes (Whatman). Membranes were incubated with the indicated primary antibodies, followed by horseradish peroxidase-conjugated goat anti-rabbit or anti-mouse secondary antibodies. Protein bands were visualized using an ECL kit (HY-K1005, MCE) on a ChemiDoc Imaging System (Bio-Rad).

### Immunofluorescence microscopy

For immunofluorescence, cells were cultured on glass coverslips prior to transfection. After transfection, cells were fixed with 4% paraformaldehyde (PFA) for 10 min at room temperature, followed by permeabilization with 0.3% Triton X-100 for 15 min. Cells were incubated overnight at 4 °C with Myc antibody (1:2,000), followed by incubation with Alexa Fluor 555-labeled Donkey Anti-Mouse IgG (H+L) (A0460, Beyotime) as the secondary antibody (Sun et al., 2021). Confocal images were acquired at room temperature using a FV1000 confocal inverted microscope and analyzed with FV10-ASW 4.2 software application (Olympus Corporation).

## Result

### Patient

The patient was a 5-year and 8-month-old female of Han nationality. She was brought to medical attention due to delayed psychomotor development compared with children of the same age. The developmental milestones were as follows: she was able to lift her head at 3 months, sit independently at 6 months, climb at 8 months, stand on her toes at 11 months, and walk at 1 year and 10 months with abnormal posture and gait. Language development included babbling at 5 months, responding to her name at 6 months, pronouncing “mom and dad” at 9 months, and speaking short sentences with unclear enunciation by 2 years of age. The patient had previously been diagnosed with “cerebral palsy (CP)” at several hospitals. From the age of 1, she underwent rehabilitation training, received neurotrophic drugs, and underwent acupuncture and other treatments, with minimal improvement. At present, at 5 years and 8 months of age, she can walk independently but with an abnormal gait and slow walking speed. The observed postural abnormalities include hip convexity, forward trunk leaning, toe dragging, and increased propensity for falls. She is able to ascend and descend stairs but cannot run, jump, or squat independently. Her speech is slow, dysarthric, and often accompanied by drooling.

**FIGURE 1:**
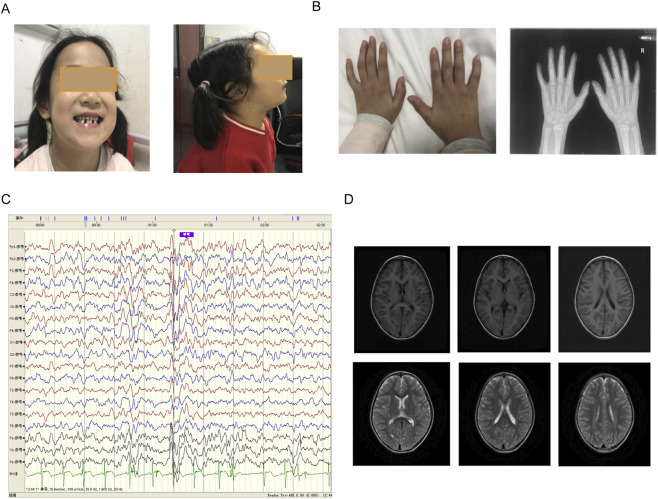
Clinical presentation of the patient. **(A)** Frontal and lateral views of the patient. **(B)** Hands and fingers of the patient. **(C)** EEG at the age of 5 years. **(D)** Brain MRI at the age of 5 years.

Since 9 months of age, she has experienced seven episodes of fever with convulsions, with body temperatures exceeding 38 °C. The seizure episodes were characterized by loss of consciousness, cyanosis of the lips, eye deviation, teeth clenching, and rigidity with shaking of the eyelids and limbs, lasting from 1 to 30 min. The patient is the second child of healthy, non-consanguineous Chinese parents and has a healthy 20-year-old sister. She was born at term via vaginal delivery without complications. The pregnancy was unremarkable, the birth weight was 3,000 g, and the Apgar scores were normal. Physical examination revealed a height of 114 cm, weight of 22 kg, head circumference of 50 cm, and chest circumference of 59 cm. She displayed good mood and frequently smiled. She was able to follow simple instructions, such as opening her mouth. Oral findings included salivation and irregular and slightly sparse teeth (Figure 1A). The tongue movements were inflexible, and speech was slow and dysarthric. Gross vision and hearing were normal; the bilateral eyeball movements were intact, pupillary light reflexes were sensitive, and pharyngeal reflex was slow. The hands exhibited slight deformities, with bent fifth fingers (Figure 1B) and mild atrophy of hand muscles. The muscle strength of the limbs was normal. The muscle tone was increased in the lower limbs. No obvious tremor was observed during the finger–nose test, and rapid alternating movements could not be performed. Postural examination revealed backward head tilt and protruded buttocks. Gait was slow and abnormal, with slight varus deformity of the feet. The sensory examination result was normal. Bilateral knee tendon reflexes were symmetrical and active, the Achilles tendons were tight, and bilateral Babinski signs were negative. Meningeal irritation signs were absent.

At 1 year and 9 months of age, video electroencephalogram (VEEG) revealed occasional brief release of irregular θ rhythms in the bilateral temporal regions. Brain MRI demonstrated slightly prolonged T1 and T2 signals near the posterior horns of the bilateral lateral ventricles. At 3 years of age, MRI examinations of the pelvis, cervical and thoracic spine, and the ankle joints showed no abnormalities. At 3 years and 6 months, the patient’s IQ assessed by the Peabody Picture Vocabulary Test (PPVT) was 84. At 4 years of age, the Gesell Developmental Schedules indicated delays in fine motor, gross motor, and speech development. At 5 years of age, VEEG during light sleep demonstrated occasional medium-amplitude sharp–slow waves and spike–slow waves in the frontal regions (Figure 1C). Brain MRI revealed long T1 and T2 signal shadows in the centrum semiovale and adjacent to the lateral ventricles, which are indicative of demyelination. At 5 years and 9 months, VEEG showed slowed background rhythm and a small number of middle-to-high-amplitude sharp waves in the right occipital and posterior temporal regions, occasionally extending to the right frontal, central, parietal, and anterior temporal regions during sleep. Brain MRI indicated symmetrical punctate and patchy long T1 abnormal signals in the bilateral basal ganglia and periventricular regions (Figure 1D). Ultrasound examinations of the heart and abdomen were normal. Routine laboratory test results, including complete blood count, urinalysis, stool analysis, biochemical profile, blood ammonia, ceruloplasmin, Epstein–Barr virus DNA, lactic acid, and homocysteine, were all within the normal limits. Metabolic disease screening revealed no abnormalities. Cerebrospinal fluid (CSF) analysis showed normal routine biochemistry, negative autoimmune encephalitis antibodies, absent oligoclonal bands, and normal intrathecal IgG synthesis rate. Electrocardiogram (ECG) indicated sinus arrhythmia. Skeletal assessment showed seven ossification centers in the carpal bones of both hands.

### Genetic analysis and identification of the *SPG20* variant

**FIGURE 2:**
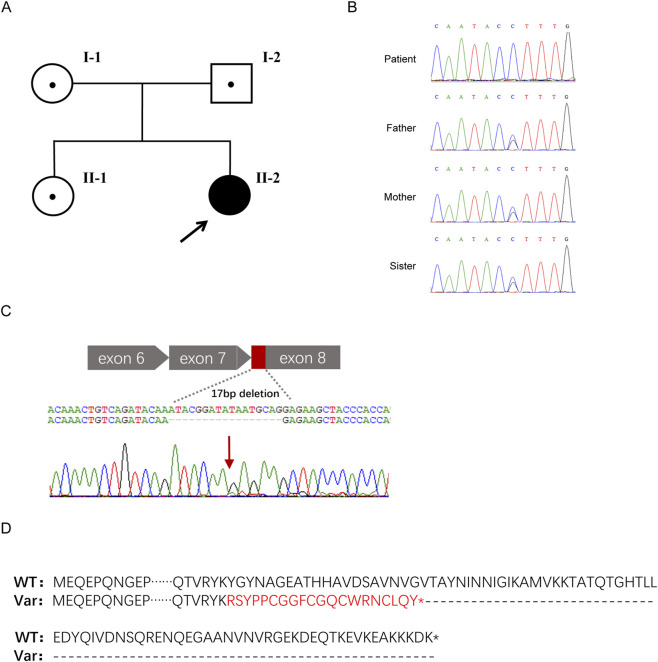
Sanger validation of the *SPG20* gene variants. **(A)** Pedigree of the patient. **(B)** Sanger sequencing validation of the *SPG20* gene variants **(C)** Spartin cDNA sequence of the patient. **(D)** Alignment of wild-type (WT) and variant (Var) spartin protein sequences.

WES revealed a homozygous pathogenic variant c.1734-1G>C in the *SPG20* gene (NM_001142294). This variant was absent in multiple population databases, including 1000 Genomes, ESP6500, ExAC, and gnomAD. Sanger sequencing confirmed the WES findings. The patient’s parents were non-consanguineous but were both heterozygous carriers of the c.1734-1G>C variant. The patient’s elder sister also carried the heterozygous variant, while all carriers (parents and sister) were asymptomatic (Figures 2A,B). To further validate the pathogenicity of this variant, mRNA was extracted from the patient’s peripheral blood, and cDNA primers were designed flanking the splice site. Sequencing of the cDNA revealed that the c.1734-1G>C variant affects splicing of exon 8 of *SPG20*, causing a 17-bp deletion at the junction of exon 8 and intron 7 (Figure 2C). This deletion causes a frameshift after amino acid 577, leading to a truncated protein of 598 amino acids (Figure 2D).

**FIGURE 3:**
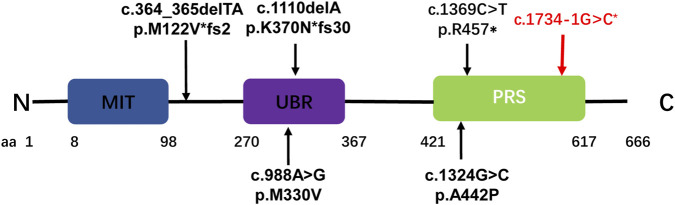
Reported variant sites of the *SPG20* gene.

This variant has not been previously reported in the literature and represents a novel variant. According to the ACMG guidelines, the c.1734-1G>C variant meets multiple pathogenicity criteria. The variant is located at a canonical splice site, fulfilling PVS1 (Figure 3). It is situated within the senescence domain of spartin, fulfilling PM1. Furthermore, this variant is absent in multiple population databases, including 1000 Genomes, ESP6500, ExAC, and gnomAD, fulfilling PM2. Therefore, this variant is classified as pathogenic (PVS1 + PM1 + PM2).

**FIGURE 4:**
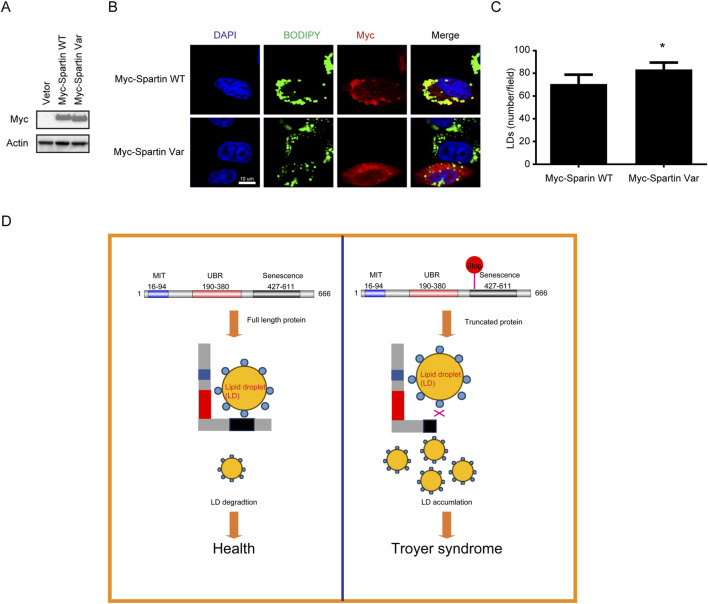
Spartin variant does not localize to lipid droplets (LDs). **(A)** Western blot analysis of lysates from HeLa cells transfected with wild-type (WT) or variant (Var) *SPG20* DNA. Uncropped images are presented in Supplementary Figure S1. **(B)** Immunofluorescence microscopy analysis of LDs and spartin localization in HeLa cells transfected with WT or mutant (Mut) spartin DNA after 0.1 mM oleic acid (OA) treatment overnight. Scale bar, 10 mm. **(C)** Total number and size of LDs were quantified in five randomly selected fields. Quantification of the number of LDs is the means ± SEM pooled from all the experiments. *p < 0.05 (unpaired two-tailed Student’s t-test). **(D)** Diagram of how the spartin variant contributes to the development of Troyer syndrome. Spartin contains three evolutionarily conserved domains: the microtubule-interacting and trafficking (MIT) domain, the ubiquitin-binding region (UBR) domain, and the plant-related senescence (PRS) domain. Variants in spartin lead to LD accumulation, which in turn promotes the development of Troyer syndrome.

Previous studies have demonstrated that the senescence domain of *SPG20* is essential for LD targeting, and truncations in this domain impair lipid transfer *in vitro* and LD turnover in cells, contributing to the pathogenesis of TRS (Eastman et al., 2009; Wan et al., 2024). To further assess the functional impact of this variant, confocal microscopy was performed in HeLa cells transfected with *SPG20* constructs. Upon staining with the LD marker BODIPY, WT *SPG20* perfectly encircled LD-positive spheres, whereas the variant *SPG20* protein was diffusely distributed and failed to localize to LDs (Figures 4A,B). Importantly, the variant *SPG20* protein increased the number of LDs (Figure 4C). These results indicate that impairment of the *SPG20* function leads to the accumulation of LDs and disrupted LD turnover, which may underlie the development of TRS in patients carrying this variant (Figure 4D).

## Discussion

In this study, we report the second case of TRS in China. Several clinical features of our patient were consistent with previously reported cases, including spastic paraplegia of the lower limbs, distal limb muscular atrophy, cognitive delay, euphoric behavior, dysarthria, mild hand deformities with small finger bending, mild cerebellar signs, and abnormal white matter, particularly in the periventricular region of the temporoparietal area. Previous reports by Butler et al. (2016) and Spiegel et al. (2017) indicated that TRS is often associated with dwarfism; however, our patient did not exhibit dwarfism. Bizzari et al. (2017) described skin abnormalities in three TRS patients, which were absent in our case.

Neuroimaging findings in TRS are heterogeneous. white matter hyperintensities in the temporoparietal periventricular regions and posterior limbs of the internal capsule in some TRS cases were reported by Proukakis et al. (2004), while other studies documented normal MRI results in certain patients (Butler et al., 2016; Spiegel et al., 2017). In our patient, brain MRI revealed symmetrical punctate and patchy long T1 signal abnormalities in the bilateral basal ganglia and periventricular regions. Such variability in MRI findings may relate to disease progression, particularly in older patients (Proukakis et al., 2004).

Convulsions have not been described in previously reported TRS cases. Our patient experienced multiple febrile seizures, characterized by transient loss of consciousness, upward gaze, and limb shaking, lasting from 1 to 30 min, with spontaneous resolution and normal postictal mental status. VEEG showed occasional medium-amplitude sharp–slow and spike–slow waves in the frontal regions during light sleep (Figure 1C). These seizures may reflect the functional impact of specific *SPG20* variant sites on spartin protein activity and neural development. The occurrence of febrile convulsions in our patient expands the clinical spectrum of TRS.

The patient was initially misdiagnosed with CP, a common diagnostic pitfall in early-onset spastic diplegia. Unlike CP, TRS is progressive and lacks a history of perinatal insult. Other complicated HSPs (e.g., SPG11, SPG15, and SPG7) and mitochondrial disorders were excluded based on neuroimaging, metabolic screening, and clinical course. The combination of progressive spastic paraplegia, dysarthria, distal muscle atrophy, and normal early cognition should prompt the consideration of *SPG20* genetic analysis.

To our knowledge, febrile seizures have not been previously reported in TRS. In our patient, recurrent febrile seizures occurred between 9 months and 5 years of age. We propose a potential mechanistic link: the c.1734-1G>C variant truncates the C-terminal plant-related senescence (PRS) domain of spartin, thus disrupting its role in mitochondrial cardiolipin binding and CoQ10 synthesis. The loss of full-length spartin may lead to complex-I deficiency and reduced ATP production. During febrile episodes, the high metabolic demand of cortical neurons may exceed this compromised energy reserve, lowering the seizure threshold. This mechanism aligns with the reversible mitochondrial defect described by Diquigiovanni et al. (2023). Clinicians should consider that febrile seizures may be part of the TRS spectrum, and patients carrying PRS-truncating variants might benefit from mitochondrial evaluation and CoQ10 supplementation.

*SPG20* encodes the spartin protein, which contains three evolutionarily conserved domains, namely, the N-terminal microtubule-interacting and trafficking (MIT) domain, which is primarily involved in intracellular protein transport; the ubiquitin-binding region (UBR) domain, which mediates lysine-63-linked ubiquitination of spartin and participates in dendritic cell aggregation; and the C-terminal PRS domain, implicated in mitochondrial targeting, LD interaction, and protein stability. In our patient, the c.1734-1G>C splice variant truncates spartin at residue 598, removing most of the PRS domain. This domain is critical for LD transfer to autophagosomes, mitochondrial targeting, and cardiolipin binding to maintain Ca^2+^ homeostasis. Consistent with this, confocal microscopy showed that the variant spartin fails to localize to LDs (Figure 4), confirming the loss of PRS-dependent lipid transfer. Interestingly, partial retention of the PRS domain (the first 22 residues) in our patient may explain the absence of short stature and skeletal deformities, which are typically observed in severe truncating variants that abolish the entire PRS domain (e.g., c.1110delA). This partial functional preservation mirrors the milder phenotype observed in the missense variant p.Gly580Arg, which is also located within the PRS domain (Dardour et al., 2017). Importantly, the presence of febrile seizures in our patient indicates that the loss of full-length spartin impairs mitochondrial protein import, decreases CoQ10 synthesis, and reduces complex-I activity, leading to ATP depletion. During fever-induced metabolic stress, cortical neurons become energetically vulnerable, lowering the seizure threshold. Thus, the PRS-truncating variants may cause predisposition to seizures, expanding the known clinical spectrum of TRS and highlighting the importance of mitochondrial assessment in *SPG20* patients.

Renvoisé et al. constructed *SPG20* knockout mice to confirm the pathogenic role of *SPG20* in TRS. They found that spartin promotes the ubiquitination of multiple LD-related proteins via atrophin-1-interacting protein 4 and regulates the biosynthesis of LD-associated proteins (Renvoisé et al., 2012). Joshi and Bakowska (2011) demonstrated that spartin regulates mitochondrial calcium homeostasis through the interaction of its C-terminal PRS domain with cardiolipin. Pathogenic variants in *SPG20* can, therefore, impair mitochondrial calcium uptake, potentially leading to axonal damage. Nahm et al. (2013) further showed in *Drosophila* that spartin controls microtubule stability and regulates synaptic development and neuronal survival via the BMP–dFMRP–Futsch pathway. Spartin is a multifunctional protein, whose functions are closely related to the development of nerve cells and the regulation of neural function. Functional variants in spartin lead to Troyer syndrome. In this patient, a homozygous c.1734-1G>C variant of the *SPG20* gene was identified. The variant in spartin leads to the accumulation of LDs, which in turn promotes the development of Troyer syndrome (Figure 4). However, the manner in which *SPG20* variants affect this process requires further functional verification.

While the majority of functional studies on *SPG20* have focused on LD homeostasis, emerging evidence indicates a critical role for spartin in mitochondrial function. Diquigiovanni et al. demonstrated that SPART loss-of-function impairs mitochondrial import of nuclear-encoded proteins, reduces levels of COQ7 and COQ9, depletes CoQ10, and consequently impairs complex-I activity and ATP synthesis (Diquigiovanni et al., 2023). In our patient, the c.1734-1G>C variant truncates the C-terminal PRS domain, which mediates spartin’s interaction with cardiolipin at the mitochondrial inner membrane (Joshi and Bakowska, 2011). The disruption of this domain compromises mitochondrial calcium uptake and membrane potential (Joshi and Bakowska, 2011). Clinically, this aligns with the patient’s febrile seizures, developmental delay, and cognitive impairment, indicating underlying mitochondrial bioenergetic dysfunction—a feature not previously emphasized in TRS. Although direct mitochondrial studies such as muscle biopsy or fibroblast respiratory chain analysis were not performed, the potential link between severe *SPG20* variants and complex-I impairment requires further investigation. If confirmed, CoQ10 supplementation could be explored as an adjunctive therapy (Diquigiovanni et al., 2023).

To date, only six pathogenic variants of *SPG20* have been reported in TRS, namely, three frameshift variants (c.1110delA p.K370N*fs30, c.364_365delAT p.Met122val*fs2, and c.892delA p.Thr298Glnfs*30) (Patel et al., 2002; Liang et al., 2020; Bakowska et al., 2008; Manzini et al., 2010), one nonsense variant (c.1369C>T p.Arg457*) (Dardour et al., 2017), and two missense variants (c.988>G p.Met330Val and c.1324G>C p.Ala442Pro) (Spiegel et al., 2017; Bizzari et al., 2017) (Figure 3). Among these, c.364_365delAT is frequently reported in TRS patients of non-Chinese ancestry, particularly in the Old Order Amish population, indicating that it is a population-specific hotspot. In contrast, the c.364_365delAT variant was not detected in this Chinese patient; instead, the novel c.1734-1G>C variant was identified and classified as pathogenic according to the ACMG guidelines.

HSP and CP share overlapping features, particularly the progressive spasticity of both lower limbs, which can lead to misdiagnosis. One of the main forms of CP is spastic diplegia, characterized by lower-limb spasticity with minimal or asymmetric involvement of the upper limbs. It is most commonly observed in preterm infants with white matter injury. In contrast, HSP typically presents as slowly progressive paralysis of the lower limbs, often accompanied by scissor gait. Some patients may also develop upper-limb involvement, dysphagia, speech disorders, and, in later stages, defecation and urinary dysfunction. The clinical similarity between CP and HSP necessitates careful differential diagnosis, and genetic testing can be a valuable tool when the diagnosis is uncertain.

To our knowledge, this study reports the second case of TRS in China, following the initial report by Liang et al. (2020). The patient carries the c.1734-1G>C variant, representing the first reported splice-site variant in the *SPG20* gene. Functional analysis at the RNA level demonstrated that this variant disrupts spartin protein function, leading to the accumulation of LDs in the overexpressing cells. This study expands the variant spectrum of *SPG20*, provides a summary of the clinical characteristics in TRS patients, and highlights correlations between the genotype and phenotype.

## Data Availability

The original contributions presented in the study are included in the article/Supplementary Material, further inquiries can be directed to the corresponding authors.
