# Supplementary figures and images for: Case Report: Novel homozygous pathogenic variant of the SPG20 gene causes the Troyer syndrome in China
Source: Front Genet. 2026 Jul 2;17:1842902. doi: 10.3389/fgene.2026.1842902 (PMC13372273; doi:10.3389/fgene.2026.1842902)

The blot for FIGURE 3A

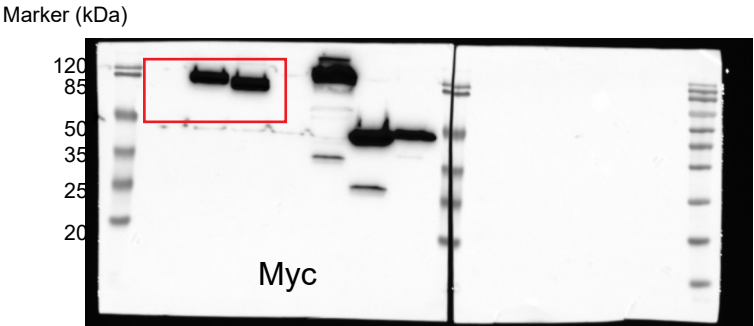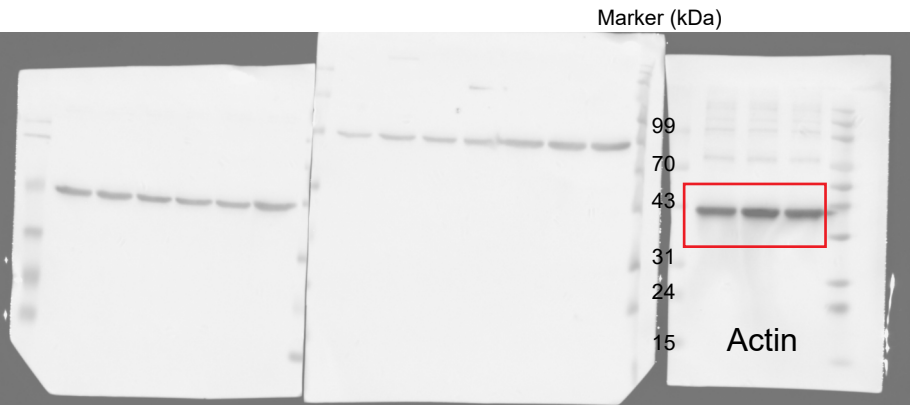

Supplement: Supplementary file 1 [file DataSheet1.pdf]
